# The Neurological Institute in New York City

**Published:** 1915-01-23

**Authors:** 


### INSTITUTIONAL ORGANISATION IN AMERICA.

### The Neurological Institute in New York City.

This hospital owes its establishment to three eminent mental specialists of New York City, Dr. Joseph Collins, Dr. Joseph Fraenkel, and Dr. Pearce A. Bailey.

The institute proved that it met a necessity of the day during the first six months of its existence, when a third of its applicants had to be turned away, as so large a number could not be dealt with. It has eighty beds, fifty in wards and thirty in private rooms for paying patients. Designed for the study and treatment of nervous diseases, no provision is made for the insane; but one fact has been impressed upon the physicians during their five years' work in the wards, and that is the uplift that many patients with chronic, and in some cases incurable, nervous diseases get from a few weeks' treatment in a hospital where they are subject to remedial measures of a non-medicinal nature. Also many cases of insanity have been averted by the prompt recognition of the gravity of certain symptoms and by the vigorous treatment adopted to overcome them.

The staff is composed of eminent specialists. Every department of medicine and surgery has for its head a name known throughout the States. The medical board includes the three founders, in addition to whom there are numerous consultants and physicians, also a neurologist, a gynæcologist, a dermatologist, an orthopædic surgeon, a laryngologist and rhinologist, an otologist, and an ophthalmologist, four surgeons, the directors of the laboratory and of the histological and neurological laboratories, an anæsthetist, and a radiologist, three chiefs of clinic, two assistant surgeons, three house physicians, a specialist in electrotherapy and his assistant, a director of massage with four assistants, a teacher of folk-dancing, another for gymnastics, an occupation teacher, and a social research worker. High salaries have been paid to ensure capable heads of these departments. The director of the laboratory reports 2,686 examinations for 1912, that is four times as many as during the first quarter. The clinical and histological laboratory, adjacent to the chemical and pathological laboratory, is the most recent addition. It is completely equipped and in full operation.

The institute in its latest report pleads for a larger building, with at least 150 beds and facilities for caring for twenty—instead of ten as at present—new patients every day. A gift of 100,000 dollars has enabled the board to realise its aim of a country branch for the after-care of patients. This will, as far as possible, be carried out on the village-colony plan, and will become a valuable educational asset; for the board believes that a neurological hospital should have every laboratory facility—chemical, pathological, psychological, and clinical—that it may profitably study and interpret the material provided.

Nervous and mental diseases require to be studied and interpreted before they can be prevented and cured. This the founders of this humane institution fully recognise—strange that England should be so lacking in the same recognition—and the vital element of their work is educational.

The department of applied therapeutics is perfectly equipped for the utilisation of water, heat, and super-heated air, electricity, Zander exercises, massage, calisthenics, re-educational movements, &c. Indeed, much of the success results from the fact that patients realise that in this hospital something tangible is being done for their relief. Almost from the outset alterations and enlargements became imperative, and were at once undertaken. Through the generosity of two ladies a roof-garden was added in the first year, where folk-dancing is practised and games are played. Two shelters admit of patients working at various arts and crafts.

Once every week the physicians working in the institute gather in conference. The rarer and more interesting cases are demonstrated and discussed, to the advantage of both medical men and patients. These conferences are open to the medical profession, and are largely attended.

The greatest increase—that of 100 per cent.—in the amount of work performed by the institute has been in the department of applied therapeutics, and a large corps of trained assistants work under a physician who gives his whole time to this one branch of healing. The occupation department has been most successful, and in a house on the roof—where patients also sleep in the summer, basket-making, weaving, sewing, knitting, embroidery, millinery, knotted-raphia work, stencilling, metal work, carpentry, drawing, modelling, and printing are carried on under the superintendence of a director. In the afternoon the patients dance.

In 1911 nine thousand clinical examinations were made, and the increase in the number of operations proves that the surgical side of the treatment of certain nervous disorders arising from spinal cord or other tumours is very important.

The social service department was established with the help of one of the trustees, in March 1912, four ladies interested in that branch of the institute providing the funds. The fundamental idea of this work is that many cases call for more intensive study and more extensive treatment than is possible during ordinary consultations. Many of the workers are volunteers, who carry the treatment into the patients' homes. The director spends much of his time in the clinics, and thus aids the physicians in preventing and combating nervous diseases, by obtaining exact and fuller information regarding the personal history and antecedents of patients than is possible during ordinary consultations, and in securing better conditions through proper agencies. There were no fewer than 425 requests for assistance in this department in about eight months.

